# The effect of the association between food budget and food quality on adherence to national guidelines in kindergartens, and the impact of budget limit on the food quality

**DOI:** 10.29219/fnr.v68.9524

**Published:** 2024-01-11

**Authors:** Kristin E. Fjæra, Ratib Lekhal, Sølvi Helseth, Milada Hagen, Samira Lekhal

**Affiliations:** 1 Faculty of Health Sciences, Oslo Metropolitan University, Oslo, Norway; 2 Department of Education; University of Oslo, Oslo, Norway; 3 GreeNudge and Morbid Obesity Center, Vestfold Hospital Trust, Oslo, Norway

**Keywords:** cross-sectional, children, kindergarten, diet, food budget

## Abstract

**Background:**

In Norway, almost 97% of children attend kindergartens. Most of the daily food intake happens during the day in kindergartens, and the quality of food and meals being served is essential to promote healthy food habits. There is variation in the food that kindergartens provide, and kindergartens can ask for additional payment from parents to cover the food. There are no rules neither across kindergartens for the food offering nor how much additional payment kindergarten can request.

**Objective:**

Our main objective is to investigate possible associations between the food budget and the quality of food offered in kindergartens. We specifically aimed to identify budget levels that were associated with better adherence to national guidelines, thereby the quality of the food provided, as recommended by the Norwegian Directorate of Health.

**Design:**

A cross-sectional study design, based on kindergarten pedagogical leaders’ answers to a web-based questionnaire.

**Settings:**

Private and public kindergartens across Norway are included in this present study.

**Participants:**

A total of 324 kindergarten staff attending on behalf of kindergartens participated.

**Results:**

The food budget thresholds over NOK 199 are associated with higher quality of served food, in adherence to national guidelines of food and meals (odds ratio 5.2, CI = [1.5, 16.5]), compared to thresholds under 199 NOK. However, increasing the monthly food budget per child to low (200–299 NOK), medium (300–399 NOK), high (400–499 NOK), or very high (>500 NOK) levels did not lead to an improvement in food quality.

**Conclusion:**

The main results reveal that budget plays a limited role in the quality of food and meals served as long as it is above ‘very low’ (199 NOK) food budget threshold. We assume that other contextual factors can influence the quality of food and meals in a more prominent role.

## Popular scientific summary

- Kindergartens play a vital role in promoting healthy eating habits and reducing social inequalities. Food budgets have been perceived as barriers to serving healthy food in kindergartens.
- The majority of children’s daily food intake occurs in kindergartens, making food quality crucial in these settings.
- We observed that the threshold for lower food quality was associated with very low food budgets (<199 NOK), but higher budget thresholds do not necessarily lead to improved food quality.
- Increasing the monthly food budget per child to low (200–299 NOK), medium (300–399 NOK), high (400–499 NOK), or very high (>500 NOK) levels did not lead to an improvement in food quality.
- Further investigation of other contextual factors that may significantly influence food quality in kindergartens is warranted.

Research has consistently shown that the early establishment of healthy habits seems to track over later in life (1). However, children’s dietary intake is inconsistent with national recommendations (2), and many children eat less fruit, berries, vegetables, whole grains, and fish than recommended (3).

Most children under school age in European countries attend kindergartens (4), and in Norway, almost 97% of children aged 1–5 attend kindergartens and spend over 41 h there weekly (5). The kindergartens in Norway are regulated by law and have a framework plan for the content and tasks, which specify that kindergartens are responsible for promoting good physical and mental health among children (6). The framework plan states that kindergarten staff should ‘Know and implement national guidelines for health promotion and preventive measures concerning children’ (6). National guidelines for food and meals in kindergartens represent one such directive that kindergartens in Norway are recommended to follow. Furthermore, kindergartens in Norway have a maximum parental payment set by the government of 3,000 NOK, which is equal both in public and private kindergartens (7). Although there is a maximum parental fee to attend kindergartens in Norway, the kindergartens can ask for an additional fee to cover food and beverages served by kindergartens. Most kindergartens ask for this (8). No legal regulations govern the additional payments, ranging from 0 to 1,200 NOK per child per month across Norwegian kindergartens (5). It remains unclear how kindergartens determine the basis for these supplementary fees for food budgets and how they are linked to the quality of food provided, as kindergartens have autonomy in making these decisions.

The national guidelines for food and meals in the kindergarten (9) explain the health authorities’ expectations for the staff’s roles and educational tasks for the meal. For instance, the national guidelines highlight that kindergartens should facilitate three meals a day and are responsible for providing variable and healthy food, which is in line with national dietary recommendations from Norwegian Directorate of Health (e.g. vegetables, fruit, whole grains, fish, low-fat dairy, soft margarine, salt, and sugar-rich foods) (9).

Nonetheless, kindergartens have the flexibility to decide whether children should bring their meals for each meal or if the kindergarten should take the responsibility of preparing and providing food. There are no comprehensive regulations governing the structure of food and meal arrangements in kindergartens. The majority of kindergartens opt to both supply and prepare one or more meals (10). We do not have any information that indicates how frequently kindergartens serve hot meals during the week. According to the most recent dietary survey of Norwegian kindergartens in 2011, 67% of children brought their breakfast, while lunch and afternoon meals were provided in 84% and 53% of the kindergartens, respectively (8). The survey also revealed that kindergartens should significantly increase the serving of vegetables, fish, and whole grains (8). A previous Norwegian study from 2021 confirmed similar results (11), which is identical internationally (12).

The food budget is often perceived as a barrier to improving the quality of food served in kindergartens (13, 14). A diet aligned with national nutrition recommendations is more costly (15–19). A review from 2016 did not find any studies related to economic factors, such as the monthly food budget and the relation to the quality of food and meals served by kindergartens (20). Still, the national survey of Norwegian kindergartens from 2011 reported that kindergarten staff stated that the monthly food budgets influence the food quality in kindergartens (8).

To the best of our knowledge, no studies have investigated a potential correlation between food budget and the quality of served food in kindergartens. Thus, our main objective is to investigate possible associations between the food budget allocated for food and the quality of food offered in kindergartens. We specifically aimed to identify budget levels associated with adherence to national guidelines, thereby quality of the food provided, as outlined by the Norwegian Directorate of Health.

## Ethics

We conducted this study according to the guidelines laid down in the Declaration of Helsinki. The participants are anonymous, and no personalized data are related to the present study.

## Methods

### Data collection

In the present study, we collected data through Healthier Kids, a free web-based programme aiming to increase theoretical knowledge and simplify practical work with food and meals. Healthier Kids tries to convey national food guidelines via e-learning modules and materials. When the kindergartens sign up, and before they get access to the web-based programmes, one pedagogic leader answers a questionnaire about their kindergarten and how they work with food and meals.

The data were collected from the recently mentioned questionnaire between March 2018 and April 2020. In this period, 374 kindergartens attended Healthier Kids and answered the web-based questionnaire. Of the 374 kindergartens, 324 ticked the box that their answers could be used for research; thus, the response rate was 86%.

### Questionnaire

The web-based questionnaire was designed to map food provided in kindergartens, based on national guidelines for food and meals in kindergartens (questions in Table 1).

**Table 1:** Questionnaire to measure quality of food served by kindergartens

*How often does your kindergartens serve fruit? *How often does your kindergartens serve vegetables? *How often does your kindergartens serve sweet beverages (fruit juice and beverages with added sugar)? *How often does your kindergartens offer sweets (cookies, waffles, and candy)? **How often does your kindergartens serve whole-grain bread? **When you serve pasta or rice in the kindergarten, how often do you serve wholegrain pasta or rice? ** When you serve milk in the kindergarten, how often do you serve diet or skimmed milk? **When you serve table butter, how often do you serve plant-based margarine, such as Soft Flora, Vita, Soft Flora Lett, Vita Lett, or Brelett, which has the ‘Keyhole’ label? **How often does your kindergartens serve fish other than fish spread, for instance cooked fish?** **‘When you serve cold cuts, does the kindergarten often serve fish cold cuts?**’ When you serve cold cuts, how often are all cold cuts marked with Keyhole?***

* The response alternatives were on a Likert scale ranging from ‘one’ (1) to ‘five days a week’ (5).

** The response alternative ranged from ‘low degree’ (1) to a ‘high degree’ (5).

*** Keyhole is a label that shows healthier products with less salt, sugar, and saturated fat and higher dietary fiber.

The questionnaire underwent testing by four pedagogical staff members from different kindergartens and one expert in the relevant field. Their feedback was incorporated into the final version of the questionnaire. It is important to note that, to the best of our knowledge, no validated questionnaires are available in the existing literature designed explicitly for assessing food and the food environment within kindergartens.

### Validation of the questionnaire

Since the questionnaire in our current study has not been validated, we performed an exploratory factor analysis using questions related to food and beverage options as recommended by national guidelines. We wanted to investigate how well it covered the underlying construct (food quality) in terms of internal consistency and reliability, so we computed Cronbach’s alpha. Furthermore, we wanted to assess if all the items loaded only on one factor or if it was possible to identify several factors (domains) that would be clinically meaningful. The factor analysis was used with orthogonal rotation (varimax), and our data revealed a four-cluster structure (Supplementary Table 1). The four clusters explained about two-thirds of the original variation in our data (the cumulative explained variance was 62.8%). The first cluster explained 28% of the variation. When we added a second cluster, it only explained an additional 14% of the original variation in our data. The third and fourth clusters only covered two items and one item, respectively. Only a few questions were loaded onto factors 3 and 4, as illustrated in Supplementary Table 1. The internal consistency was measured with Cronbach’s alpha of 0.68, indicating that these items exhibited similar trends or directions. In explorative analysis, Cronbach’s alpha between 0.6 and 0.7 is considered acceptable (21). The rationale for keeping only one domain was that the third and fourth factors were less robust, covering only two and one items, respectively.

Therefore, considering the limited explanatory power of the additional clusters and the acceptable internal consistency, we decided not to consolidate the questionnaire into distinct domains and instead focus on the overarching domain measuring the overall quality of food served that aligns with our research objectives.

### Measurement

#### Descriptive

We collected demographic information, the monthly food budget per child, whether children brought their lunch boxes or if the kindergartens provided food for some or all meals each day, and the number of children in both toddler (ages 1–3) and pre-K (ages 3–5) classroom.

#### Variable quality of served food in kindergartens

The variable describes the variety of food and beverages served daily in the kindergarten and is composed of 11 questions. These questions are listed in Table 1. In the present study, the quality of served food refers to whether kindergartens adhere to the national guidelines and recommendations from the Norwegian Directorate of Health, which indirectly measures food quality in this study.

The score includes 11 questions about healthy foods (eg. fruits and vegetables) and unhealthy foods (eg. sweets). For unhealthy foods, the points in the score were weighted inverse: the lower the frequency of offering, the higher the score.

Before calculating a total score for overall quality (here defined as adherence to the national guidelines), each question was answered using a scale ranging from 1 to 5. These scores were further dichotomized to 0 (original score 1–3) and 1 (original score 4–5).

The overall score was then summarized, ranging from 0 to 11. The study sample was subsequently divided into two groups. Kindergartens with a score above eight were categorized as serving food with higher quality, indicating that they served meals in line with national guidelines. Conversely, a score below eight suggested that kindergartens did not meet the quality standards outlined in the recommended guidelines.

#### Independent variable food budget

We assessed the monthly food budget as the parental additional monthly payment for food and beverages per child, determined by the kindergarten, assessed with an open-ended question; ‘What is the additional monthly food budget per child in your kindergarten?’ Furthermore, we re-coded the responses into one variable, with five different categories: 0) very low food budget (0–199 NOK), 1) low food budget (200–299 NOK), 2) medium food budget (300–399 NOK), 3) high food budget (400–499) NOK, and 4) very high food budget (>500 NOK).

### Statistical method

All statistical analyses were performed using the statistical software SPSS® Statistics version 27.0. Continuous variables are described with median and range and categorical variables as counts and proportions.

We used logistic regression to explore possible associations between the level of the food budget and the quality of food served by kindergartens. The outcome variable is dichotomous, while the independent variable has five categories (very low, low, middle, high, and very high food budget levels). We assume that the number of meals could affect the monthly food budget (i.e. additional parental payment for food and beverage) compared to kindergartens where children bring one or several meals from home. Therefore, we adjusted for this in the analysis. Additionally, a department with a higher frequency of children might influence meals provided since the additional food budget is higher. Therefore, we also adjusted for this.

The reference category in the primary analysis was a very low food budget (Table 2).

**Table 2:** Quality of food served in kindergartens and associations with various food budget thresholds (*n* = 299)

Food budget	OR	95% CI	*P*-value	*N*
Very low food budget (ref)			0.070	26
Low food budget	5.1	1.5–16.5	0.006	62
Medium food budget	3.5	1.1–11.0	0.028	125
High food budget	4.8	1.3–15.9	0.014	58
Very high food budget	4.7	1.4–16.0	0.012	53
Meals a day			0.001	
**Sensitivity analysis with higher budget threshold (low = 200–299 NOK)**				
Low food budget (ref)				62
Medium food budget	0.68	0.34–1.3	0.269	125
High food budget	0.92	0.37–2.2	0.858	58
Very high food budget	0.59	0.28–1.2	0.161	53
Meals a day			0.001	

Logistic regression. Reference group = very low food budget 0–199 NOK, adjusted for the total number of meals a day.

Very low food budget level (0–199 NOK), low food budget level (200–299 NOK), medium food budget level (300–399 NOK), high food budget level (400–499 NOK), and very high food budget level (500–599 NOK).

Sensitivity analysis with higher threshold budget (low = 200–299 NOK).

7% missing data.

Cutoff score, dependent variable = 8.

We tested the strength of the possible association between the low-budget level and the middle, high, and very high budget levels and the food quality. In addition, we performed a sensitivity analysis with the outcome (i.e. quality of food served) and dichotomized it using a different cutoff than the one used in the primary analyses. We additionally did a sensitivity analysis when we excluded two questions to measure the frequency of serving sweet beverages and cookies and explore if this will influence the results.

The results are reported as odds ratios (ORs) with 95% confidence intervals (CIs). We considered all analyses exploratory, so we did not perform any correction for multiple testing, and we considered *P*-values < 0.5 as statistically significant. All tests were two-sided.

## Results

### Descriptive results

From 2018 to 2020, 324 kindergartens from different areas in Norway answered the questionnaire.

Data in the present study are based on responses from 324 kindergartens, of which 68.5% are private and 31.5% are public. Furthermore, 155 kindergartens were from eastern Norway, 41 from mid-Norway, 50 from the west, 48 from the south, and 26 from northern Norway.

The average number of meals offered daily by kindergartens was 2.3, which means that most of the kindergartens in the present study prepared and provided food for the meals. This reveals that few kindergartens asked children to bring their food in a lunch box for the meals. The median number of children attending the toddler classroom was 23.08, and the pre-K classroom was 41.04. The private kindergartens had a median food budget of 350 NOK, whereas this figure for public kindergartens was 280 NOK.

### Associations between the quality of food served by kindergartens and the different levels of the food budget

The food budget thresholds over NOK 199 are associated with a higher quality of food and meals served by kindergartens (OR = 5.1, 95% CI = [1.5, 16.5]) (Table 2). However, we did not observe a linear increase in the quality of food served by kindergartens when the food budget per child was higher (Table 2). The odds for high food quality remained similar for low, middle, high, and very high budgets compared to the very low ones (Table 2).

These findings suggest that a very low food budget is likely to have a negative impact on the quality of food and meals. However, the association between a higher food budget and improved food quality appears unclear. Interestingly, even with a very high or medium budget, there was no noticeable increase in food quality (Table 2). There was no significant difference between public and private kindergartens (not shown in tables) (*P* = 0.14).

### Sensitivity analysis testing different levels of additional food budget per children

We performed a sensitivity analysis to investigate whether there were any variations in food quality among different budget levels when the reference category was set as a low budget. Our analysis found no statistically significant associations between the different budget levels and food quality when the reference was a low budget. These findings were similar to what we found in the primary analysis (Table 2).

### Sensitivity analysis with quality of food served with a lower cutoff in measured food quality than in the primary analysis

We performed a sensitivity analysis using a lower cutoff in the quality score (from a score of 8 down to 7 of 11) (Supplementary Table 2). The results showed that only the lowest budget groups were associated with the quality of food served by kindergartens, similar to what we found in the primary analysis (Table 2).

### Sensitivity analysis on quality of food served, excluding questions about sweet beverages and cookies in the variable ‘food quality’, and association with food budget

We performed an additional sensitivity analysis by excluding two questions concerning sweet beverages and cookies (as shown in Table 1). We did an analysis with very low and low-budget groups as the reference category. In this revised analysis, we established a new criterion for assessing high quality, requiring kindergartens with a score of more than five to meet the quality standards in accordance with national guidelines. The outcomes of this supplementary analysis (as presented in Supplementary Table 3) remained consistent with the results of our primary analysis (Table 2).

## Discussion

The present study aimed to explore to which degree there is an association between the food quality in adherence to national guidelines, and how low the budget limit is before it affects food quality.

The main results reveal that budget plays a limited role in the quality of food and meals served as long as it is above a minimum limit – this is surprising and contrary to assumptions from previous research. For instance, research to identify perceived implementation difficulties and barriers to achieving best practice standards among kindergarten staff has shown that 81% of them mean that the food budget and food cost are barriers to serving high-quality food (22). Other studies have indicated the same (13, 14, 23). A cluster-randomized study from Norway, intending to investigate possible associations between vegetable intake and a size of food budget indicated that kindergartens with a higher food budget (>251 NOK) served more vegetables than those with a food budget below 251 NOK (24). The same study also found that kindergartens with budget lower than 251 NOK served less vegetables in total but tended to have a greater variety of vegetables. Consequently, the authors inferred that factors beyond budget considerations, such as environmental or sociocultural factors, likely played a role in influencing the number of vegetable servings (24). Even though the referred study only explored vegetables, and not overall food quality, it is, nonetheless, relevant in light of our study because it highlights the significance of other factors within the kindergarten environment that influence food serving practices.

Finnish pre-schools have specific nutrition guidelines; the food and meals served in these kindergartens are included in maximal parental payment (25). Despite this, Finnish pre-schools do not meet the recommended healthy food serving, especially fruit and vegetables (16). The fact that food budgets are included does not automatically ensure that the quality of food served in kindergartens aligns with the Finnish nutrition guidelines. This supports the idea that other factors, such as the kindergarten environment, could influence what is served (25, 26). This is interesting in light of the results of the current study. Mainly since the monthly food budget per child across kindergartens in Norway ranges from 0 to 1,200 NOK (27), in the present study, it ranges from 120 to 1,000 NOK. The absence of limits on food budgets and food offerings across kindergartens underscores the importance of exploring additional factors. Furthermore, even within the same municipality or kindergarten chain, kindergartens providing all daily meals may not maintain consistent monthly food budgets per child. Denmark (26) and Norway have both additional monthly parental payments for food and beverages per child, and the results in the present study might be of extra importance in countries that practice the same standard (26).

The fact that the monthly food budget did not increase in line with the quality of food can be explained, similar to previous research (24, 25), with other factors that could influence the quality of food provided by kindergartens (25). Previous research suggested that staff knowledge and attitudes toward healthy food, policy, and menu planning may have an impact on food serving (23, 28). Although we did not observe a definitive enhancement in food quality with increasing food budgets, it is important to note that this lack of a clear correlation does not necessarily negate a direct relationship. It does, however, raise some interesting points to consider. Suppose one believes that parents’ monthly additional fee is the most crucial factor for food quality in kindergartens. This has the potential to exacerbate social inequalities, since Norwegian kindergartens have the autonomy to set their fees, making it a political and governmental issue which should be lifted. According to WHO, social inequalities regarding health must be solved in rural and rich countries (29). Reducing social differences in health is important according to general public health and is, therefore, highlighted in Norway in the public health law (30). Children from families with low social status often eat more unhealthy and are less physically active than others (31). Children from different social classes attend kindergartens; therefore, kindergartens are important for promoting healthy habits and reducing social inequalities (32). In order to provide equal opportunities for all children, it is crucial to ensure uniformity in the quality of food and meals served in Norwegian kindergartens. We advocate for a more comprehensive exploration of additional factors within the kindergarten environment to prevent the imposition of unnecessarily high monthly food expenses per child, which may contribute to heightened social inequalities.

### Strengths and limitations of the study

One of the key strengths of this study is its focus on an under-researched sector in Norway, namely, kindergartens. Furthermore, the present study brings new reflection on how one should emphasize food budget as one of the most crucial factors related to the quality of food that kindergartens serve in the future. Suppose we carefully interpret the results of this study, in that case, it may indicate that other factors beyond the food budget also play a more important role in what is served in the kindergartens. The web-based questionnaire was not validated but tested for reliability, so we performed a factor analysis to investigate its structure and internal validity. The Cronbach’s alpha was 0.68, which is considered acceptable (21). The question ‘How often is diet milk served in the kindergarten?’ loaded low (<0.1), and when we excluded this item, Cronbach’s alpha increased to 0.78. Nevertheless, we performed the primary analysis with this item (milk) included because milk is essential in Norwegian kindergartens and included in the dietary guidelines. There are certain limitations to the questionnaire. Specifically, we did not inquire about the frequency of weekly hot meal provision in kindergartens. We believe this omission is not expected to impact the outcomes significantly, but we will include a question regarding the frequency of hot meals being served in our future research.

A further limitation of our study is that we did not measure the amount of meat and meat products (e.g. red or white meat), which we should include in future research.

However, it is a strength that we did measure whether the kindergarten used the keyhole label when selecting spread (e.g. fish, meat, liver-paste, etc.). This is not mentioned in the guidelines but indicates that kindergartens choose healthier spreads than other options in the same food category.

There is a limitation in how we created the variable measuring food quality. We lacked previous diet scores for comparison. The variable we used in this study, the quality of food served, draws some inspiration from the Healthy Eating Index (HEI) score (33). It is important to note that many studies have employed various tools and scoring systems to assess diet quality (34, 35). However, these tools are typically designed for evaluating individual dietary intake. In this study, we focus on assessing the quality of food served in kindergartens concerning the recommendations from national guidelines rather than evaluating the dietary quality of children’s food consumption. While this study does not provide a comprehensive description of food quality, we believe that the different food assessments included in the overall quality score indicate whether the food served in kindergartens aligns with national guidelines for food and meals. The score used in this study is an indirect measure of food quality. Another aspect to have in mind is the potential variation in how ‘food quality’ is interpreted, which could make comparing our findings with other studies challenging. Nevertheless, it is reasonable to assume that the definition of food quality in the context of kindergartens is relatively consistent, especially when referring to the food served in kindergartens. Another hurdle to be aware of when making comparisons with other studies is the alignment of national guidelines with terms used in international literature, like ‘guidelines’, ‘policy’, and ‘dietary recommendations’.

According to statistics in Norway, the proportions of public and private kindergartens are 47% and 53%, respectively. In the present study, 68.5% of kindergartens were private, and 31.5% were public; thus, our sample will likely be subjected to a selection bias. Our sample represents about 5.8% of Norway’s total number of kindergartens. Therefore, combined with the selection bias mentioned earlier, it limits the generalizability of our results. In the present study, kindergartens from different regions of the country contribute to a greater sample diversity, but it does not necessarily guarantee its representativeness. Of the 374 kindergartens, 324 answered web-based questionnaires from 2018 to 2020. This gives a response rate of 85%, according to those who participate in Healthier Kids. The data analyzed come from Healthier Kids, ‘a free web-based program aiming to increase theoretical knowledge and simplify practical work with food and meals’. Therefore, the studied kindergartens could be more committed to promoting healthy eating than the other kindergartens.

The CIs of our estimates were broad and reflected limited precision; thus, our results must be interpreted cautiously.

Furthermore, the web-based questionnaire was answered by pedagogical leaders or leaders who responded to questions on behalf of the kindergarten staff. Unfortunately, this could be a limitation as answers may not reflect the actual practice in kindergartens. However, we assumed that pedagogical leaders or leaders were the best representatives to give answers on how the overall work with food and meals for their kindergarten.

It might be a weakness that we are not entirely sure if kindergartens use their own funds, thereby increasing the overall food budget. Still, they ask for additional payment, which we have recorded in the present study. In Norway, it is common for kindergartens to request additional payment from parents for food in addition to the maximum fee. Therefore, we assume it is relevant to explore whether a high or low food budget is associated with a higher likelihood that kindergartens serve food in line with recommendations from the Health Directorate and further follow the national guidelines or not. However, our results indicate that other factors within kindergartens in addition to the size of the food budget seem to be important regarding food serving.

### Implication for further research

Because there are variations in the food quality served in Norwegian kindergartens (8, 11) and other countries (26, 36), there is a need to explore possibilities that can streamline work with food and meals. In addition, the results from this study might add new perspectives that there could be facilitators other than food budgets influencing the quality of food served in kindergartens, which should be explored in additional research.

## Conclusion

This study illustrates that the quality of food served in alignment with national guidelines was influenced when the budget threshold was below 199 NOK. There was no evident improvement in food quality with higher budget thresholds. This underscores the need to further investigate other contextual factors that could exert a substantial influence on the quality of food and meals in kindergartens.

## Authorship

I confirm that all the authors have contributed to the main manuscript. F.K. drafted the manuscript and performed data analysis with support from one of the cowriters. M.S., L.R., H.S., and L.S. edited the manuscript. S.M. double-checked the statistics in the manuscript. All authors have critically revised the manuscript and read and approved the final version.

## Ethical standards disclosure

This study was conducted according to the guidelines in the Declaration of Helsinki. Regional Committee approved all procedures involving research study participants for Medical and Health Research Ethics and the Norwegian Centre for Research. A written informed consent was obtained from all subjects. We confirm that all analyses and data collection were performed per relevant guidelines and regulations. Furthermore, Oslo Metropolitan University and the Research Council of Norway have approved the protocol/project description about the research project and what we aim to explore.

The participants in this research project are at the institutional level, and our data are de-identified. In this study, participants answered questionnaires on behalf of the institution. We confirmed that informed consent was obtained from all subjects in the project. There is a plan for publishing all project results after receiving funding from the Research Council of Norway.

## Data Availability

The data are stored in Greenudge in a remote access solution, and the files will only be used for this project. All information is stored and de-identified. The dataset used and analyzed during the current study is available upon request. Please contact the PI of this study, Samira Lekhal: samira@greenudge.no, for further information.
