# Supplementary material for: The effect of the association between food budget and food quality on adherence to national guidelines in kindergartens, and the impact of budget limit on the food quality
Source: Food Nutr Res. 2024 Jan 11;68:10.29219/fnr.v68.9524. doi: 10.29219/fnr.v68.9524 (PMC10845897; doi:10.29219/fnr.v68.9524)
Supplement: Supplementary file 1 [file FNR-68-9524-s001.docx]

**Supplementary tables**

**Table I- Factor analysis**

|  | Factor 1 | Factor 2 | Factor 3 | Factor 4 |
| --- | --- | --- | --- | --- |
| How often does your kindergartens serve fruit?  How often do your kindergartens serve vegetables?  How often does your kindergarten serve whole-grain bread?  When you serve cold cuts, how often does your kindergarten serve fish-cold cuts?  How often does your kindergarten serve fish?  When you serve pasta or rice in the kindergarten, how often do you serve whole-grain rice and pasta?  When you serve cold spread, how often are the cold cuts marked with a keyhole?  When your kindergarten serve milk, how often do you serve skimmed and diet milk?  When you serve table butter, how often does your kindergarten serve margarine (e.g. Soft Flora, Vita)?  How often does your kindergarten serve sweet beverages including juice  How often does your kindergarten serve sweets (cookies, waffles)? | **0.57**  0.22  **0.74**  **0.75**  0.08  0.25  **0.65**  0.05  **0.67**  −0.02  0.03 | 0.19  **0.62**  0.26  0.20  **0.79**  **0.74**  0.01  0.02  0.06  0.09  −0.07 | −0.25  −0.22  −0.06  0.00  0.16  −0.01  0.09  0.03  0.08  **0.85**  **0.84** | 0.12  0.22  −0.12  −0.12  0.05  −0.21  0.23  **0.94**  0.04  0.01  0.02 |

Factor analysis of variable quality of served food in kindergartens.

**Table II- Association between the quality of served food with a lower cut-off (score >7) and different levels of the food budget (n=299).**

| Food budget | OR | 95 % CI | p-value |
| --- | --- | --- | --- |
| Very low food budget (ref) |  |  | .238 |
| Low food budget | 2.9 | 1.0-8.2 | .043 |
| Medium food budget | 2.5 | .89-7.0 | .080 |
| High food budget | 3.4 | .95-12.7 | .059 |
| Very high food budget | 1.8 | .60-5.9 | .274 |
| Meals a day  Toddler  Pre-k classroom  Priv/public |  |  | .001  .746  .181  .196 |

*Logistic regression. Reference group =0-199 NOK. Adjusted for the total number of meals a day, frequency of children in toddler and pre-k classroom.

*7 % missing

*Cut-off score, outcome =7

**Table III- Logistic regression quality of food served (excluding questions regarding sweet beverages and sweet cookies etc) associated with food budget.**

| Food budget | OR | 95 % CI | p-value |
| --- | --- | --- | --- |
| Very low food budget (ref) |  |  | .196 |
| Low food budget | 3.0 | 1.0-8.9 | .040 |
| Medium food budget | 2.2 | .8-6.5 | .121 |
| High food budget | 3.6 | 1.0-11.9 | .037 |
| Very high food budget | 3.1 | 1.00-9.9 | .049 |
| Meals a day | 1.8 | 1.2-2.5 | .001 |
| Reference category low food budget (200-299 NOK) | | | |
| Low food budget (ref) |  |  | .394 |
| Medium food budget | .76 | .36-1.3 | .391 |
| High food budget | 1.05 | .44-2.4 | .912 |
| Very high food budget | 1.3 | .48-3.9 | .555 |
| Meals a day | 2.0 | 1.3-3.0 | .001 |
| *Logistic regression. Reference group =0-199 NOK and 200-299 NOK. Adjusted for the total number of meals a day.  *7 % missing  *Cut-off score, outcome =5  *Excluding two items (sweet beverages and sweet cakes) in the total score | | | |
